# Serum Biomarkers for Discrimination between Hepatitis C-Related Arthropathy and Early Rheumatoid Arthritis

**DOI:** 10.3390/ijms18061304

**Published:** 2017-06-19

**Authors:** Isabela Siloşi, Lidia Boldeanu, Viorel Biciuşcă, Maria Bogdan, Carmen Avramescu, Citto Taisescu, Vlad Padureanu, Mihail Virgil Boldeanu, Anica Dricu, Cristian Adrian Siloşi

**Affiliations:** 1 Department of Immunology-Laboratory of Immunology, University of Medicine and Pharmacy of Craiova, 2 Petru Rares Street, Craiova 200349, Romania; isabela_silosi@yahoo.com; 2 Department of Microbiology, University of Medicine and Pharmacy of Craiova, 2 Petru Rares Street, Craiova 200690, Romania; barulidia@yahoo.com (L.B.); c.avramescu@yahoo.com (C.A.); 3 Medico Science SRL-Stem Cell Bank Unit, 1B Brazda lui Novac Street, Craiova 200690, Romania; 4 Department of Internal Medicine, University of Medicine and Pharmacy of Craiova, 2 Petru Rares Street, Craiova 200690, Romania; biciuscaviorel@gmail.com (V.B.); taisescu@yahoo.com (C.T.); vldpadureanu@yahoo.com (V.P.); 5 Department of Pharmacology, University of Medicine and Pharmacy of Craiova, 2 Petru Rares Street, Craiova 200349, Romania; bogdanfmaria81@yahoo.com; 6 Department of Functional Sciences, University of Medicine and Pharmacy of Craiova, 2 Petru Rares Street, Craiova 200690, Romania; anica.dricu@live.co.uk; 7 Department of Surgery, Faculty of Medicine, University of Medicine and Pharmacy of Craiova, 2 Petru Rares Street, Craiova 200349, Romania; cristian_silosi@yahoo.fr

**Keywords:** hepatitis C virus-related arthritis, early rheumatoid arthritis, interleukin 6, tumor necrosis factor-α

## Abstract

In the present study, we aimed to estimate the concentrations of cytokines (interleukin 6, IL-6, tumor necrosis factor-α, TNF-α) and auto-antibodies (rheumatoid factor IgM isotype, IgM-RF, antinuclear auto-antibodies, ANA, anti–cyclic citrullinated peptide antibodies IgG isotype, IgG anti-CCP3.1, anti-cardiolipin IgG isotype, IgG anti-aCL) in serum of patients with eRA (early rheumatoid arthritis) and HCVrA (hepatitis C virus-related arthropathy) and to assess the utility of IL-6, TNF-α together with IgG anti-CCP and IgM-RF in distinguishing between patients with true eRA and HCVrA, in the idea of using them as differential immunomarkers. Serum samples were collected from 54 patients (30 diagnosed with eRA-subgroup 1 and 24 with HCVrA-subgroup 2) and from 28 healthy control persons. For the evaluation of serum concentrations of studied cytokines and auto-antibodies, we used immunoenzimatique techniques. The serum concentrations of both proinflammatory cytokines were statistically significantly higher in patients of subgroup 1 and subgroup 2, compared to the control group (*p* < 0.0001). Our study showed statistically significant differences of the mean concentrations only for ANA and IgG anti-CCP between subgroup 1 and subgroup 2. We also observed that IL-6 and TNF-α better correlated with auto-antibodies in subgroup 1 than in subgroup 2. In both subgroups of patients, ROC curves indicated that IL-6 and TNF-α have a higher diagnostic utility as markers of disease. In conclusion, we can say that, due to high sensitivity for diagnostic accuracy, determination of serum concentrations of IL-6 and TNF-α, possibly in combination with auto-antibodies, could be useful in the diagnosis and distinguishing between patients with true eRA and HCV patients with articular manifestation and may prove useful in the monitoring of the disease course.

## 1. Introduction

Chronic hepatitis diseases have multifactorial etiology. The experimental data and clinical observations have shown that the prevalence of chronic liver infections with hepatitis C virus (HCV) is about 3% [1]. Besides the primary effects manifested in the liver, chronic HCV infection may be associated with various extrahepatic manifestations (approximately 40–70%), such as arthralgias, arthritis, vasculitis, paresthesia, myalgia, pruritus, sicca syndrome, cryoglobulinemia and glomerulonephritis. Hence, we ought to differentiate between chronic HCV infection and primitive rheumatic disease [2,3].

Studies have shown that the prevalence of hepatitis C virus-related arthropathy (HCVrA) is about 4% of patients presenting with HCV. This is a small percentage because many patients are diagnosed as having an articular event only when consulting a specialist. Polyarthritis, similarly to early rheumatoid arthritis (eRA), is symmetrical, involving mainly small joints but not associated with articular bony erosions [4]. Poanta L. et al. undertook a prospective study in which presented evidence that 20% of patients infected with HCV will have arthralgia in first year [5]. It has been observed that articular manifestations present in patients with HCV are rheumatoid arthritis type or arthritis associated with deposits of cryoglobulines. These patients have a high prevalence of positive rheumatoid factor (RF) and therefore can often be wrongly diagnosed with eRA [6].

Because both eRa and HCVrA are complex disorders with multifactorial etiology, in clinic, diagnosis problems may occur. One of the diagnosis problems is represented by the differentiation of eRA from HCVrA: the signs and symptoms of HCVrA, including joint involvement, can be similar to eRA, making the distinction between HCVrA and eRA difficult [7,8]. Moreover, the presence of erosions, rheumatoid nodules, or positive anti–cyclic citrullinated peptide antibodies (anti-CCP) in a patient, should generate the possible diagnosis of eRA. It is possible for HCV patients to develop nonerosive arthritis without nodules, and it can also imitate eRA, so, if the erosions are not observed, the discrimination is unable to be made easily [9,10].

The objective of this study was to estimate the concentrations of cytokines (interleukin 6, IL-6 and tumor necrosis factor-α, TNF-α) and auto-antibodies (rheumatoid factor IgM isotype, IgM-RF, antinuclear auto-antibodies (ANA), anti-CCP antibodies IgG isotype, IgG anti-CCP3.1, anti-cardiolipin IgG isotype, IgG anti-aCL) in serum of patients with eRA and HCVrA and to assess the utility of IL-6, TNF-α together with IgG anti-CCP and IgM-RF in distinguishing between patients with true eRA and HCVrA, with the idea of using them as differential immunomarkers.

## 2. Results

### 2.1. Clinical Characteristics of the Study Subjects

Among the 30 patients initially diagnosed with eRA (subgroup 1), 80% were female (sex ratio: 24 female/6 male), with age, mean ± stdv 55.77 ± 10.87 years. In the HCVrA subgroup (subgroup 2), a female to male ratio was 14/10 (67%), mean age was 54.42 ± 7.49 years. In the control group, incidence for women was 78% and age 52.36 ± 13.38 years. There was no significant difference in age between the two subgroups (*p* = 0.342) (Table 1).

### 2.2. Cytokines Concentrations

In our study, we found that both proinflammatory cytokines IL-6 (38.77 pg/mL, 95% CI: 26.93–50.60) and TNF-α (63.32 pg/mL, 95% CI: 45.39–81.26) concentrations in the serum of patients in subgroup 1 were higher than those in the control group (2.85 pg/mL, 95% CI: 2.08–3.62, *p* < 0.0001 and 4.29 pg/mL, 95% CI: 3.33–5.25, *p* < 0.0001, respectively). We also found statistical differences between serum concentrations of IL-6 (29.13 pg/mL, 95% CI: 20.01–38.24) and TNF-α (54.63 pg/mL, 95% CI: 36.50–72.76) of patients in subgroup 2 and the control group (*p* < 0.0001) (Table 2).

**Figure 1:**
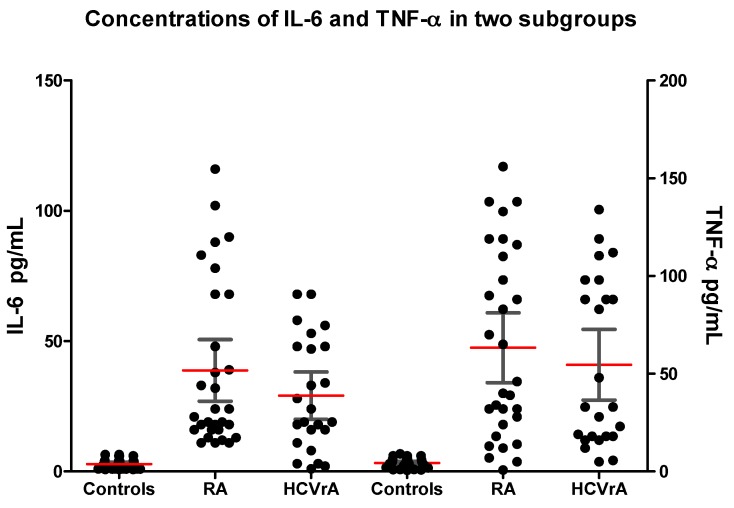
Interleukin 6 (IL-6) and tumor necrosis factor-α (TNF-α) concentrations in serum of patients in both subgroups and control group (Black circles represent IL-6 and TNF-α concentration of individual serum samples; red lines represent mean values accompanied by 95% confidence interval; 95% confidence interval is represented by black horizontal bars).

When we compared the mean concentrations of IL-6 and TNF-α between the two subgroups, we noticed that we have no statistically significant differences (*p* = 0.388, respectively *p* = 0.481) (Figure 1).

In the studied cohort of patients, we observe statistically significant differences in the concentrations of CRP and the levels of ESR between both subgroups and the control group (CRP/control group-*p* < 0.0001, ESR/control group-*p* < 0.0001) (Table 1). Comparing the mean concentrations of CRP and ESR between the two subgroups, we noticed that we have highly statistically significant differences between subgroup 1 and subgroup 2 (*p* < 0.0001).

### 2.3. Auto-Antibodies Concentrations

Another objective of our study was to investigate auto-antibodies profile in both subgroups. In Table 2, we reproduced concentrations of these auto-antibodies investigated.

Following the analysis, our study showed statistically significant differences of the mean concentrations only for ANA and IgG anti-CCP between subgroup 1 and subgroup 2 (ANA, subgroup 1/subgroup 2-*p* = 0.006, respectively IgG anti-CCP subgroup 2/subgroup 1-*p* < 0.0001) (Table 2).

### 2.4. Correlations Between IL-6, TNF-α and Auto-Antibodies in eRA and HCVrA

Concentrations of both cytokines are correlated with each other in subgroup 1 (*r* = 0.337, *p* = 0.049) and not correlated in subgroup 2 (*r* = −0.154, *p* = 0.471) (Table 3 and Table 4). In addition, we observed that IL-6 and TNF-α better correlated with auto-antibodies in subgroup 1 than in subgroup 2.

In subgroup 1, we noticed that: IL-6 correlated fairly well with IgG anti-CCP (*r* = 0.418, *p* = 0.044), TNF-α correlated with IgG anti-aCL (*r* = 0.349, *p* = 0.050) and CRP (significant negative correlation, *r* = −0.404, *p* = 0.027) and IgM-RF correlated with IgG anti-CCP (*r* = 0.418, *p* = 0.022) and IgG anti-aCL (significant negative correlation, *r* = −0.320, *p* = 0.049). 

In subgroup 2, we observe that: IL-6 correlated fairly well with IgM-RF (*r* = 0.578, *p* = 0.003), TNF-α correlated with IgG anti-aCL (significant negative correlation, *r* = −0.411, *p* = 0.046), ANA correlated with IgG anti-CCP (*r* = 0.428, *p* = 0.037) and IgG anti-aCL (strong positive correlation, *r* = 0.649, *p* < 0.0001) and IgG anti-CCP correlated with IgG anti-aCL (strong positive correlation, *r* = 0.694, *p* < 0.0001).

### 2.5. Diagnostic Performance of IL-6 and TNF-α as Disease Markers

Comparing the ROC curves for the studied parameters in the two subgroups of patients indicated that IL-6 and TNF-α have a higher diagnostic utility as markers of disease (Table 5).

**Figure 2:**
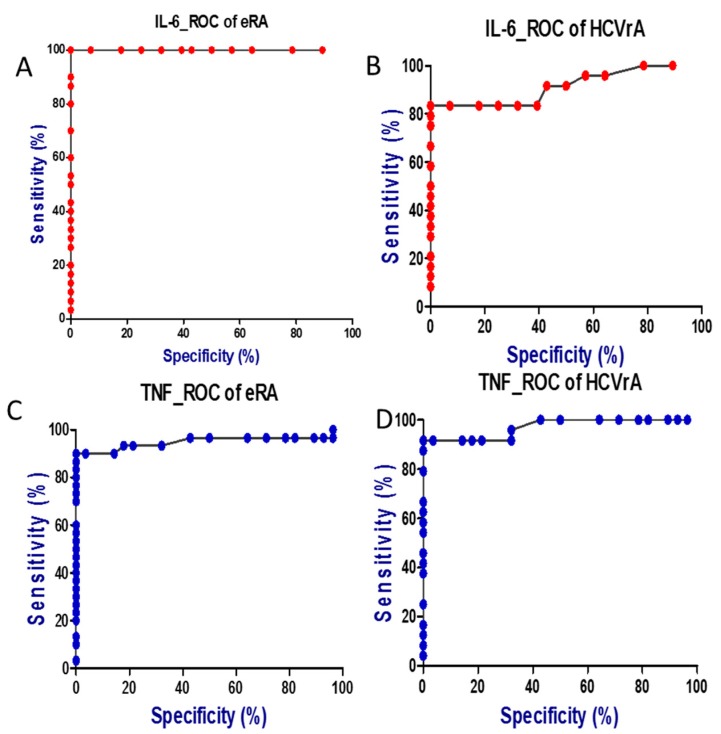
Comparison of receiver operating characteristic (ROC) curves for IL-6 and TNF-α in two subgroups of patients. (**A**) ROC curve for IL-6 in eRA subgroup; (**B**) ROC curve for IL-6 in HCVrA subgroup; (**C**) ROC curve for TNF-α in eRA subgroup; (**D**) ROC curve for TNF-α in HCVrA subgroup.

ROC analysis revealed that IL-6 concentration indicated eRA presence with 100% accuracy using the concentration of 8.75 pg/mL as an optimal cut-off value for discrimination between patients with eRA and controls (95% CI: 1.000–1.000, *p* < 0.0001). The likelihood ratios of positive and negative results obtained on the basis of optimal threshold values specific for eRA were as follows: LR(+) = 14.00 and LR(−) = 1.12 with sensitivity and specificity equal to 100 and 100%, respectively, Youden index was 1.000 (Figure 2).

ROC analysis revealed that the IL-6 concentration indicated HCVrA presence with 97.50% accuracy using the concentration of 7.25 pg/mL as an optimal cut-off value for discrimination between patients with eRA and controls (95% CI: 0.927–1.024, *p* < 0.0001). The likelihood ratios of positive and negative results obtained on the basis of optimal threshold values specific for eRA were as follows: LR(+) = 11.67 and LR(−) = 1.12 with sensitivity and specificity equal to 100% and 96.43%, respectively, the Youden index was 0.964.

In case of TNF-α the calculated cut-off value for discrimination between patients with eRA and controls was 10.50 pg/mL and using this value the diagnostic accuracy of TNF-α was 95.00% (95% CI: 0.883–1.017, *p* < 0.0001). The likelihood ratios of positive and negative results obtained on the basis of optimal threshold values specific for TNF-α were as follows: LR(+) = 25.20 and LR(−) = 1.04 with sensitivity and specificity equal to 90.00% and 100%, respectively, the Youden index was 0.900.

The cut-off value of TNF-α for discrimination between patients with HCVrA and controls was 10.50 pg/mL and using this value the diagnostic accuracy of TNF-α was 97.10% (95% CI: 0.929–1.013, *p* < 0.0001). The likelihood ratios of positive and negative results obtained on the basis of optimal threshold values specific for TNF-α were as follows: LR(+) = 25.67 and LR(−) = 1.04 with sensitivity and specificity equal to 91.67% and 100%, respectively, the Youden index was 0.917.

## 3. Discussion

Rheumatoid arthritis (RA) is a chronic, progressive, systemic inflammatory autoimmune disease in which the body’s immune system mistakenly attacks the joint. The disease produces an inflammatory infiltrate of immune cells as well as a series of destructive events such as: synovial hyperplasia, pannus setting, bone and cartilage erosion and joint destruction. It results in swelling and pain in the joints and around them [11,12].

In serum and synovial fluid collected from patients with RA we can determine besides RF and anti–citrullinated protein antibodies (aCCPs), numerous auto-antibodies involved in the disease etiopathogenesis [13,14]. Circulating auto-antibodies (ANA; anti-smooth muscle antibody, ASMA; anti–mitochondrial antibody, AMA) have been implicated in about 48% of Chinese patients with chronic HCV infection [15]. In the study made by Buskila D. et al., the authors remark that RF (44%), ANA (38%), IgM anti-aCL (28%) and IgG anti-aCL (22%) were predominant, in cases with HCV-positive patients [16]. ANA represents a serologic biomarker that is useful for diagnosing patients with autoimmune or connective tissue diseases.

The two groups studied by us had similarly low levels of ANA. We noted smaller ANA incidences in groups of investigated patients (16% in HCVrA, respectively 25% in eRA group).

According to Narciso-Schiavon J.L. et al., ANA positivity can constitute an immunological epiphenomenon, on addressing the HCV patients [17]. It has been reported that up to 20% of healthy people have positive ANA [18]. Unlike the general population, the prevalence of anti-aCL antibodies is higher in patients with chronic HCV infection [19]. Various studies have reported that chronically infected HCV patients have low titers of ASMA, RF, anti-liver-kidney-microsomal antibodies (aLKM), ANA and anti-aCL antibodies [20,21]. Patients with chronic HCV infection may have a high prevalence of IgG isotype anti-aCL (22%) [22]. In HCV infection, Ordi-Ros J et al. found that the frequency of anti-aCL was lowered by 6.6% [23].

In our study we observed that the frequency of anti-aCL antibodies in HCVrA infection was 3%. Many research studies reported that, in comparison to the general population, the anti-aCL antibodies prevalence is higher in HCV infection [2,6,24].

RFs (all three isotypes IgM, IgA and IgG) represent the auto-antibodies, discovered initially in RA [25]. Involvement of isotype IgM-RF was observed in most studies with high titres and appearing in the initial stages of development of RA [26,27].

Here, we found that, in the serum level of a positive IgM-RF, the eRA concentration was higher than the HCVrA concentration, but the result was not statistically significant (*p* = 0.276), this parameter being useless in the differential diagnosis.

The eRA and HCV patients with articular manifestation showed a 79% and 64.7% IgM-RF [28]. There might be 50–70% of cases with RF positive with HCV infection where patients display rheumatic symptoms and signs [29]. The study carried out by Sène D. et al. showed that RF positivity was around 81% on patients with RA, whereas, in HCVrA, this positivity was situated between 54–82% [30]. 

The majority of researchers [16,31,32,33] were able to identify the antibodies IgM-RF in eRA (between 70% and 80%) and nonartritic HCV infected patients (19–80%), the same way as we identified them in HCVrA patients, who displayed noteworthy incidence (70%). Owing to the fact that there could be seen a similarity in both the prevalence of positive RF in investigated patients with HCVrA, and with arthritis, the test is not to be used to make a reliable distinction between this current condition and the classic eRA, a case mentioned by other researches too [9,33,34].

Because of the low specificity of RF, in many clinical trials, new serological markers were identified to contribute with good diagnostic accuracy to the diagnosis of patients with eRA. One of these serological markers are anti-CCP auto-antibodies. Nishimura K. et al. achieved a specificity of 95% for anti-CCP and concluded that anti-CCP antibody had a better diagnostic accuracy than RF in the diagnosis of patients with eRA [35]. In patients that have eRA, the increased specificity of 98% of anti-CCP antibody might constitute a reason to eliminate other rheumatic or immune diseases in patients with positive anti-CCP [35,36,37,38,39,40,41]. Pinheiro GC et al. acknowledged that anti-CCP antibodies are more specific for RA (96–98%) and present in nearly 75% to 80% of patients with RA [42].

In our study, anti-CCP was positive in 66.6% of patients with eRA and respectively in 12.5% of HCVrA patients. We found that anti-CCP antibodies have approximately equal specificity and sensitivity (96.43% respectively 96.67%) in eRA, unlike patients with HCVrA where we met a specificity of 100% and a lower sensitivity (83.33%).

The presence of anti-CCP antibodies is certified, by some studies, in 4.5% to 7% of the HCVrA patients [30,34]. A different study showed that 83% of patients with established eRA and 4.5% of patients with HCVrA had the anti-CCP antibody [24,43]. The patients infected with HCV are diagnosed differentially for arthritis, due to the specific anti-CCP positivity, and this is more significant for eRA than the other causes [44].

Our HCVrA and eRA patients presented an interesting prevalence of the positivity for anti-CCP, 2/24 (8%) and 20/30 (66.6%) respectively, but without significant correlation between such parameters like ANA, IgM-RF, IL-6, TNF-α and articular involvement. There were no correlations between the IgM-RF and the IgG anti-CCP in HCVrA. This finding was also mentioned by other authors (*r* = 0145; *p* = 0498) [33], unlike eRA where IgG anti-CCP correlated fairly well with IgM-RF (*r* = 0.418; *p* = 0.022) and IL-6 (*r* = 0.371; *p* = 0.044). These data provide strong evidence that the specificity recently attributed to this parameter in the diagnosis of RA is meaningful. In order to make a distinction between antibodies and cyclic citrullinated peptide for discriminating eRA of HCV infection, serological tests are used, due to the fact that the antibodies proved to be positive in patients with eRA only [33].

In the immune response to viral agents, a significant part is played by cytokines [45]. Cytokines are known to be the key mediators of inflammation and joint destruction in RA [46,47]. IL-6, known as a pro–inflammatory cytokine, is usually elevated during acute infection and inflammation [48,49].

In the present work, serum levels of IL-6 in both subgroups, the eRA and HCVrA, were significantly higher than that of the control group (*p* < 0.001). The patients with HCVrA had lower values compared to the patients with eRA levels. The enhanced serum levels of IL-6 in eRA patients indicate an increased synthesis and hyperactivity of this cytokine in eRA. The increased values of this cytokine in the RA and HCVrA patients suggests common synthesis mechanisms.

There were authors who noticed increased IL-6 level in RA and HCVrA [50]. Chung S.J. et al. found significantly elevated levels of IL-6 in the serum of patients with RA, correlated with CRP levels and in patients with severe disease activity it was observed that concentrations of IL-6 and IL-11 decreased with improvement of symptoms. The authors concluded that these results suggest the involvement of IL-6 in the pathogenesis of RA, with IL-6 levels reflecting disease activity [51].

Various pro-inflammatory cytokines seem to be activated in chronic HCV infection, the progress of chronic hepatitis C being attributed mostly to them [52]. TNF-α is one of the best-studied cytokines involved in HCV infection [53], TNF-α is considered to be a target in eRA, and the effect of anti–TNF-α monoclonal antibody therapy in eRA is subject to debate [54,55].

The findings of our studied cases offer interesting suggestions regarding the role of cytokines in pathogenesis of HCV complicated with arthritis. Patients with chronic HCV infection and arthropaty had a higher level of circulating TNF-α compared to those without articular implication (*p* < 0.0001). The involvement of some cytokines in the pathogenesis of eRA has also been demonstrated in another study already published by our research team. We have shown the involvement of IL-13 and IL-17 in pathogenesis of eRA, serum concentrations of IL-13, IL-17, anti-CCP and IgM-RF were statistically significantly higher in patients with eRA, compared to the controls and we concluded that IL-13 and IL-17 might be of better use in the prediction of eRA activity status than IgM-RF and anti-CCP [56].

We demonstrated that the concentrations of the studied cytokines (IL-6, TNF-α) in serum better correlated with the eRA indices than the HCVrA indices. Concentrations of both cytokines are correlated with each other in subgroup 1 (*r* = 0.337, *p* = 0.049) and not correlated in subgroup 2 (*r* = −0.154, *p* = 0.471). We also observed that IL-6 and TNF-α better correlated with auto-antibodies in subgroup 1 than in subgroup 2. The correlations between IL-6 and TNF-α were not very high.

When evaluating diagnostic utility of IL-6, TNF-α and auto-antibodies (IgM-RF, ANA, IgG anti-CCP, IgG anti-aCL), their performances in terms of both diagnostic accuracy and Youden index are comparable with the notion that IL-6 and TNF-α have higher specificity and sensitivity in both subgroups. In the previous study we showed that IL-13 has a higher diagnostic utility than IL-17, CRP, ESR, IgM-RF and anti-CCP as markers of disease activity [56].

In conclusion we can say that, due to high sensitivity for discrimination/diagnostic accuracy, determination of serum concentrations of IL-6 and TNF-α, possibly in combination with auto-antibodies could be useful in the diagnosis and distinguishing between patients with true eRA and HCV patients with articular manifestation and may prove useful in the monitoring of the disease course. In the future, in a study that will continue on this, we propose to apply this method to another cohort of the patients, consisting of more subjects, to check this model. We also propose to use another method and compare it to that used in this study.

## 4. Materials and Methods

### 4.1. Subjects and Clinical Assessment

The study group consisted of 54 patients (30 patients diagnosed with eRA-ubgroup 1, gender ratio 6 M/24 F, mean age 56.22 years; and 24 patients diagnosed with HCVrA-subgroup 2, gender ratio 10 M/14 F, mean age 54.42 years). In parallel, we investigated a control group that included 28 persons unaffected by eRA and HCVrA. Controls were matched for sex, age at the time point of blood sampling, and area of residence (rural or urban).

Early RA patients fulfilled the American College of Rheumatology (ACR) 1987 revised criteria for the classification of RA [57]. They were all investigated, diagnosed and included into the studied group, following the revised classification criteria of the American College of Rheumatology in 2010 [58]. All patients fitted the inclusion criteria for eRA (two or more swollen joints dating from more than 2 weeks, but less than 12 months from onset).

The patients with chronic HCV infection were included if there was a history of HCV seropositivity (HCV antibodies) confirmed by PCR for the RNA HCV detections. The evidence of persistent infection was established by liver biopsy or abnormal transaminases.

All patients co-infected with HIV or other hepatitis viruses (patients with positive HBs Ag) and affected by diseases with similar clinical features, particularly psoriatic arthritis, systemic lupus erythematosus (SLE), Sjögren’s syndrome, dermatomyositis, or overlap syndromes such as mixed connective tissue disease, have been excluded from the study. Those subjects who received antiviral or biological agents within 6 months of baseline were excluded too.

Serum samples were collected from 54 patients and from 28 controls (healthy persons) and analyzed for concentrations of cytokines IL-6 and TNF-α, ANA, IgG anti-CCP3.1, IgM-RF, IgG-aCL, erythrocytes sedimentation rate (ESR) and C-reactive protein (CRP).

### 4.2. Samples Collection

Blood samples were obtained from all subjects in tubes without additives by venous puncture in a fasting state in the morning. Peripheral venous blood was collected into separator vacutainers and allowed to clot for 30 min at room temperature. The test tubes were centrifuged at 3000× *g* for 10 min, and serum samples were further divided into aliquots and stored at −80 °C until assessment. Before testing, frozen probes were brought to room temperature, avoiding freezing-unfreezing cycles.

### 4.3. Immunological Investigations

Serological profile of patients with eRA, HCVrA and controls was performed in the Immunology Laboratory of University of Medicine and Pharmacy of Craiova.

The analysis of serum parameters was based on a quantitative sandwich ELISA, according to the manufacturer’s instructions. Serum ANA, IgG Anti-CCP3.1 and IgG-aCL were determined by ELISA, using Quanta Lite^TM^-INOVA Diagnostics kits, (San Diego, CA, USA) (auto-antibodies seropositivity was defined according to the manufacturer’s instructions). The investigation of serum IgM-RF concentrations was achieved using AESKU.Diagnostics GmbH ELISA kits (Wendelsheim, Germany) (positive > 15 U/L). For hsCRP dosage we used a DRG International, Inc. ELISA kit (Springfield, NJ, USA) (the positive values were > 10 mg/L). 

Serum concentrations of proinflammatory cytokines IL-6 and TNF-α were measured in patients and in control persons using PeliKine^TM^ human ELISA kit (Amsterdam, The Netherlands).

Cryoglobulin was done to all patients. For the identification of cryoglobulins presence, 10 mL of blood was drawn from fasting patients and allowed to clot and the serum was then separated at 37 °C. The samples were stored at 4 °C for 2 weeks and the cryoprecipitate presence was noted. Positive samples were then rewarmed to 37 °C to determine if the cryoprecipitate re-dissolved.

All the procedures were followed in accordance with the ethical standards of the institutional responsible committees for human studies and with the Helsinki Declaration of 1975, as revised in 2008. For realization of this study, we obtained the approval of the Committee of Ethics and Academic and Scientific Deontology of the University of Medicine and Pharmacy from Craiova number 76/2014.

### 4.4. Statistical Analysis

Patients’ data, management system, and data processing were performed using Microsoft Excel and the Data Analysis module; statistical analysis was done using GraphPad Prism 5 Trial Version (San Diego, CA, USA). All tests were two-sided and *p* values ≤ 0.05 were considered significant.

The significance of differences between groups was examined with a Mann-Whitney U test or Kruskal-Wallis, when multiple comparisons were made. Correlation analysis between the concentration of proinflammatory cytokines IL-6 and TNF-α and concentration of some auto-antibodies (ANA, IgG Anti-CCP3.1, IgG-aCL and IgM-RF), CRP and ESR, were conducted with a Pearson's test. All tests were two-sided and *p* values ≤ 0.05 were considered significant. 

The diagnostic values of studied markers were evaluated using receiver operating characteristic (ROC) curves analysis. The performance was expressed as the area under the ROC curve (AUC, area under ROC curve) together with 95% confidence interval (95% CI) and *p* statistics for the difference between calculated AUC and AUC = 0.5 (weak discriminative marker). Cut-off values corresponding to the highest accuracy were determined and for various threshold values investigated at each marker, we calculated the sensitivity (Sn), specificity (Sp), and Youden index (sensitivity + specificity −1).

## 5. Conclusions

In conclusion, we can say that, due to high sensitivity for diagnostic accuracy, determination of serum concentrations of IL-6 and TNF-α, possibly in combination with auto-antibodies, could be useful in the diagnosis and distinguishing between patients with true eRA and HCV patients with articular manifestation and may prove useful in the monitoring of the disease course.

## Figures and Tables

**Table 1:** Clinical characteristics of the study subjects.

Character	eRA Subgroup 1 (*n* = 30)	HCVrA Subgroup 2 (*n* = 24)	*p* Value	Controls (*n* = 28)
Age (years) (mean ± stdv)	55.77 ± 10.87	54.42 ± 7.49	*p* = 0.342	52.36 ± 3.38
Gender (female /male)	24/6 (80%)	14/10 (67%)	−	22/6 (78.5%)
CRP (mg/dL)	16.97 ± 5.14	16.27 ± 8.24	*p* < 0.0001	4.75 ± 2.15
*ESR* (mm/1st h)	33.60 ± 12.35	15.71 ± 6.33	*p* < 0.0001	11.68 ± 6.24
Cryoglobulinemia	13.33%	28%		

**Table 2:** IL-6, TNF-α and auto-antibodies (antinuclear auto-antibodies—ANA, anti–cyclic citrullinated peptide antibodies IgG isotype, IgG anti-CCP, anti-cardiolipin IgG isotype, IgG anti-aCL, rheumatoid factor IgM isotype, IgM-RF) concentrations in serum of patients with eRA, HCVrA and in the control group.

Parameter (Mean, 95% CI)	Levels in Subgroup 1			Levels in Subgroup 2			Levels in Subgroups		
	RA	Control	*p*	HCVrA	Control	*p*	RA	HCVrA	*p*
IL-6 (pg/mL)	38.77 (26.93–50.60)	2.85 (2.08–3.62)	< 0.0001	29.13 (20.01–38.24)	2.85 (2.08–3.62)	*< 0.0001*	38.77 (26.93–50.60)	29.13 (20.01–38.24)	= 0.388
TNF-α (pg/mL)	63.32 (45.39–81.26)	4.29 (3.33–5.25)	< 0.0001	54.63 (36.50–72.76)	4.29 (3.33–5.25)	*< 0.0001*	63.32 (45.39–81.26)	54.63 (36.50–72.76)	= 0.481
ANA (U/mL)	12.43 (9.35–15.52)	4.55 (3.84–5.28)	< 0.0001	17.67 (15.76–19.58)	4.55 (3.84–5.28)	*< 0.0001*	12.43 (9.35–15.52)	17.67 (15.76–19.58)	= 0.006
IgG anti-CCP (U/L)	100.40 (69.45–131.30)	5.75 (4.34–7.16)	< 0.0001	16.99 (14.92–19.07)	5.75 (4.34–7.16)	*< 0.0001*	100.40 (69.45–131.30)	16.99 (14.92–19.07)	< 0.0001
IgM-RF (U/L)	65.27 (45.71–84.83)	5.07 (3.95–6.19)	< 0.0001	44.42 (31.88–56.96)	5.07 (3.95–6.19)	*< 0.0001*	65.27 (45.71–84.83)	44.42 (31.88–56.96)	= 0.276
IgG anti-aCL (U/mL)	13.97 (11.19–16.74)	5.82 (4.41–7.24)	< 0.0001	16.12 (12.85–19.38)	5.82 (4.41–7.24)	*< 0.0001*	13.97 (11.19–16.74)	16.12 (12.85–19.38)	= 0.346
CRP (mg/dL)	16.97 (15.06–18.89)	4.75 (3.92–5.59)	< 0.0001	16.27 (12.79–19.75)	4.75 (3.92–5.59)	*< 0.0001*	16.97 (15.06–18.89)	16.27 (12.79–19.75)	< 0.0001
ESR (mm/1st h)	33.60 (28.99–38.21)	11.68 (9.47–14.31)	< 0.0001	15.71 (13.04–18.39)	11.68 (9.47–14.31)	*< 0.0001*	33.60 (28.99–38.21)	15.71 (13.04–18.39)	< 0.0001

**Table 3:** Correlations between IL-6, THF-α and early rheumatoid arthritis (eRA) indices.

Parameter	ANA	IgG Anti-aCL	IgM-RF	IgG Anti-CCP	IL-6	TNF-α	CRP	ESR
ANA		*r* = −0.069 *p* = 0.717	*r* = 0.273 *p* = 0.145	*r* = −0.157 *p* = 0.407	*r* = −0.026 *p* = 0.890	*r* = 0.076 *p* = 0.688	*r* = 0.251 *p* = 0.181	*r* = 0.089 *p* = 0.639
IgG anti-aCL			*r* = −0.320 *p* = 0.049 *	*r* = 0.052 *p* = 0.784	*r* = 0.158 *p* = 0.460	*r = 0.349* p = 0.050 *	*r* = 0,129 *p* = 0.496	*r* = 0.274 *p* = 0.142
IgM-RF				*r* = 0.418 *p* = 0.022 *	*r* = −0.231 *p* = 0.219	*r* = −0.131 *p* = 0.492	*r* = 0.294 *p* = 0.115	*r* = 0.071 *p* = 0.709
IgG anti-CCP					*r* = 0.371 *p* = 0.044 *	*r* = −0.231 *p* = 0.219	*r* = −0.039 *p* = 0.837	*r* = −0.005 *p* = 0.979
IL-6						*r* = 0.337 *p* = 0.049 *	*r* = 0.017 *p* = 0.928	*r* = 0.029 *p* = 0.877
TNF-α							*r = −*0.404 *p* = 0.027 *	*r* = 0.112 *p* = 0.557
CRP								*r* = −0.020 *p* = 0.916

*r* Pearson correlation coefficient, * Statistically significant correlations.

**Table 4:** Correlations between IL-6, THF-α and hepatitis C virus-related arthropathy (HCVrA) indices.

Parameter	ANA	IgG Anti-aCL	IgM-RF	IgG Anti-CCP	IL-6	TNF-α	CRP	ESR
ANA		*r* = 0.649 *p* < 0.0001*	*r* = 0.077 *p* = 0.721	*r* = 0.428 *p* = 0.037 *	*r* = −0.202 *p* = 0.345	*r* = −0.160 *p* = 0.456	*r* = −0.037 *p* = 0.864	*r* = 0.303 *p* = 0.149
IgG anti-aCL			*r* = 0.070 *p* = 0.744	*r* = 0.694 *p* < 0.0001 *	*r* = −0.056 *p* = 0.794	*r* = −0.411 *p* = 0.046*	*r* = 0.140 *p* = 0.515	*r* = −0.050 *p* = 0.817
IgM-RF				*r* = −0.203 *p* = 0.498	*r* = 0.578 *p* = 0.003*	*r* = −0.052 *p* = 0.809	*r* = −0.269 *p* = 0.204	*r* = 0.210 *p* = 0.325
IgG anti-CCP					*r* = −0.054 *p* = 0.802	*r* = −0.122 *p* = 0.571	*r* = 0.185 *p* = 0.386	*r* = −0.139 *p* = 0.516
IL-6						*r* = −0.154 *p* = 0.471	*r* = −0.101 *p* = 0.640	*r* = 0.298 *p* = 0.158
THF-α							*r* = 0.050 *p* = 0.816	*r* = 0.015 *p* = 0.944
CRP								*r* = −0.342 *p* = 0.101

*r* Pearson correlation coefficient, * Statistically significant correlation.

**Table 5:** Diagnostic performance of the investigated parameters.

Parameter		AUC Accuracy	Cut-off Value	*p* Value	Sensitivity %	Specificity %	Youden Index
RA	IL-6	1.000	8.75	<0.0001	100.00	100.00	1.000
	TNF-α	0.950	10.50	<0.0001	90.00	100.00	0.900
	IgG anti-CCP	0.982	11.50	<0.0001	96.67	96.43	0.931
	ANA	0.798	9.00	0.0001	63.33	100.00	0.633
	IgM-RF	0.991	9.50	<0.0001	100.00	96.43	0.964
	IgG anti-aCL	0.824	11.50	<0.0001	60.00	92.86	0.529
HCVrA	IL-6	0.975	7.25	<0.0001	100.00	96.43	0.964
	TNF-α	0.971	10.50	<0.0001	91.67	100.00	0.917
	IgG anti-CCP	0.914	7.25	<0.0001	83.33	100.00	0.833
	ANA	1.000	9.20	<0.0001	100.00	100.00	1.000
	IgM-RF	0.935	15.00	<0.0001	87.50	96.43	0.839
	IgG anti-aCL	0.891	11.50	<0.0001	79.17	92.86	0.720
